# Inducible Loss of the Aryl Hydrocarbon Receptor Activates Perigonadal White Fat Respiration and Brown Fat Thermogenesis via Fibroblast Growth Factor 21

**DOI:** 10.3390/ijms20040950

**Published:** 2019-02-22

**Authors:** Nathaniel G. Girer, Dwayne Carter, Nisha Bhattarai, Mehnaz Mustafa, Larry Denner, Craig Porter, Cornelis J. Elferink

**Affiliations:** 1 Department of Pharmacology and Toxicology, The University of Texas Medical Branch at Galveston, Galveston, TX 77555, USA; ngirer89@gmail.com (N.G.G.); dwaynecarter1981@gmail.com (D.C.); mehnazmustafa.mehnaz3@gmail.com (M.M.); 2 Division of Surgical Sciences, Department of Surgery, UTMB & Metabolism Unit, Shriners Hospitals for Children, The University of Texas Medical Branch at Galveston, Galveston, TX 77555, USA; nibhatta@utmb.edu (N.B.); cr2porte@utmb.edu (C.P.); 3 Division of Rehabilitation Sciences, School of Health Professions, The University of Texas Medical Branch at Galveston, Galveston, TX 77555, USA; 4 Department of Internal Medicine, The University of Texas Medical Branch at Galveston, Galveston, TX 77555, USA; ladenner@utmb.edu

**Keywords:** aryl hydrocarbon receptor, fibroblast growth factor 21, uncoupling protein 1

## Abstract

The aryl hydrocarbon receptor (AHR) is a ligand-activated transcription factor highly expressed in hepatocytes. Researchers have employed global and liver-specific conditional *Ahr* knockout mouse models to characterize the physiological roles of the AHR; however, the gestational timing of AHR loss in these models can complicate efforts to distinguish the direct and indirect effects of post-gestational AHR deficiency. Utilizing a novel tamoxifen-inducible AHR knockout mouse model, we analyzed the effects of hepatocyte-targeted AHR loss in adult mice. The data demonstrate that AHR deficiency significantly reduces weight gain and adiposity, and increases multilocular lipid droplet formation within perigonadal white adipose tissue (gWAT). Protein and mRNA expression of fibroblast growth factor 21 (FGF21), an important hepatokine that activates thermogenesis in brown adipose tissue (BAT) and gWAT, significantly increases upon AHR loss and correlates with a significant increase of BAT and gWAT respiratory capacity. Confirming the role of FGF21 in mediating these effects, this phenotype is reversed in mice concomitantly lacking AHR and FGF21 expression. Chromatin immunoprecipitation analyses suggest that the AHR may constitutively suppress *Fgf21* transcription through binding to a newly identified xenobiotic response element within the *Fgf21* promoter. The data demonstrate an important AHR-FGF21 regulatory axis that influences adipose biology and may represent a “druggable” therapeutic target for obesity and its related metabolic disorders.

## 1. Introduction

The aryl hydrocarbon receptor (AHR) is a ligand-activated transcription factor within the Per-ARNT-SIM protein family that regulates the physiological and pathophysiological responses to polycyclic and halogenated aromatic hydrocarbon exposure [1]. Unliganded AHR exists in the cytosol bound to a protein complex consisting of heat shock protein 90, X-associated protein 2 (also known as AIP or ARA9), and p23. Upon ligand binding, this complex is shuttled into the nucleus and the AHR dissociates to form a heterodimer with its DNA binding partner, aryl hydrocarbon receptor nuclear translocator (ARNT). The AHR-ARNT heterodimer then binds to the xenobiotic response element (XRE), defined by the consensus 5′-GCGTG-3′ motif, within the promoter region of target genes to activate transcription.

Recent studies have linked the AHR to the transcriptional regulation of fibroblast growth factor 21 (FGF21) [2,3,4]. FGF21 is an important hepatokine that induces thermogenesis in white and brown fat deposits by uncoupling mitochondrial respiration from the electron transport chain [5]. This activity primarily occurs via uncoupling protein 1 (UCP1) [6]. FGF21 has also been implicated in the browning of white fat deposits, a process characterized by a reduction in adipocyte size, the formation of multilocular lipid droplets, and increased thermogenesis [5,7]. Although FGF21 displays autocrine activity in certain tissues, endocrine-acting FGF21 protein appears to exclusively originate from the liver [8,9]. Exogenous FGF21 administration counteracts obesity in several different animal models [10,11,12,13], and clinical trials have already been conducted in obese human patients utilizing an FGF21 variant, LY2405319 [14,15]. Homeostatic AHR function appears to have an inhibitory role in modulating *Fgf21* transcription, given that AHR loss consistently results in elevated hepatic *Fgf21* gene expression [2,3]. However, the precise role of the AHR in regulating *Fgf21* transcription remains unclear, as exogenous AHR agonists can also activate *Fgf21* transcription [2].

Efforts to define a physiological role for hepatic AHR have employed the constitutive, hepatocyte-targeted AHR conditional knockout mouse model (*Ahr^fl/fl^Alb-Cre*, herein referred to as “cCKO”) [16]. In this study, we utilized cCKO mice to investigate the role of hepatic AHR in energy homeostasis. We observed that female cCKO mice, exclusively, exhibit reduced adiposity and increased multilocular lipid droplet formation within perigonadal white adipose tissue (gWAT). Because AHR deletion occurs gestationally in the cCKO mice, efforts to differentiate the primary effects of AHR deletion in adulthood from the potential secondary effects caused by a persistent loss of the AHR throughout growth and development prove difficult. Therefore, we generated an inducible AHR conditional knockout mouse model (*Ahr^fl/fl^Alb-Cre^ERT2^*, abbreviated “iCKO”) that expresses a modified *Cre* recombinase, allowing for the timed induction of AHR loss in adult mice following tamoxifen treatment. Similar to cCKO female mice, female iCKO mice display reduced adiposity and increased multilocular lipid droplet formation in gWAT. Given the documented ability of FGF21 to induce the browning of white fat [5] and previous data demonstrating that AHR loss induces hepatic FGF21 production [3], we hypothesized that increased FGF21 expression may account for the brown adipose tissue-like phenotype observed in gWAT collected from cCKO and iCKO female mice. To address this hypothesis, we generated an inducible, double-conditional AHR-FGF21 knockout mouse model (*Ahr^fl/fl^Fgf21^fl/fl^Alb-Cre^ERT2^*, abbreviated “DKO”) that concomitantly eliminates hepatocyte AHR and FGF21 expression in response to tamoxifen treatment. We also presented evidence that AHR binding to distinct XREs within the *Fgf21* promoter region in response to the agonist 2,3,7,8-tetrachlorodibenzo-*p*-dioxin (TCDD) is nuanced, given that both loss of the receptor and activation by TCDD can induce FGF21 expression in the liver. Together, the data presented here indicate that the AHR can influence adipose biology via an AHR-FGF21 regulatory axis in the liver.

## 2. Results

### 2.1. Constitutive Loss of Hepatocyte AHR Expression in Female Mice Reduces Body Mass and Adiposity under Standard Feeding Conditions and High-Fat Diet Challenge

**Figure 1:**
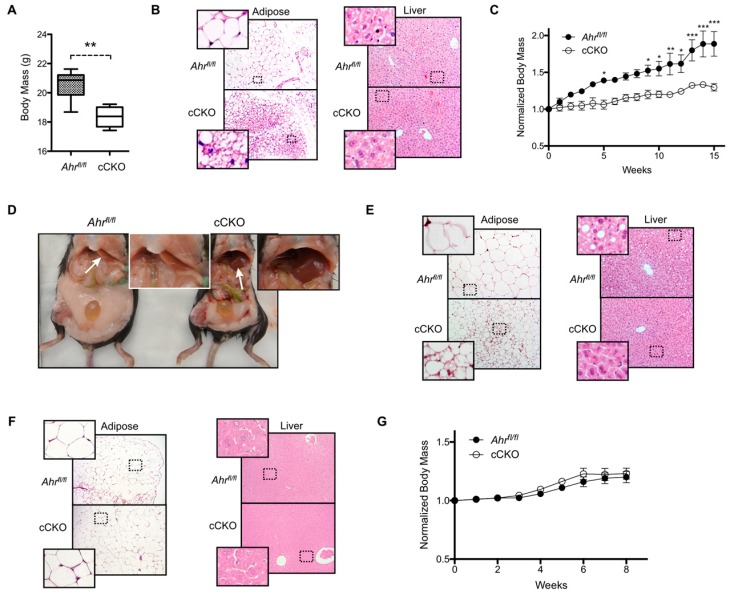
Constitutive loss of hepatocyte aryl hydrocarbon receptor (AHR) expression in female mice reduces weight gain and adiposity. (**A**) 10-week old female hepatocyte-targeted AHR conditional knockout mice (*Ahr^fl/fl^Alb-Cre*, herein referred to as “cCKO”) exhibit significantly decreased body mass (*n* = 6 per genotype). (**B**) Hematoxylin and eosin (H&E)-stained perigonadal white adipose tissue (gWAT) shows increased multilocular lipid droplet formation in 10-week old female cCKO mice relative to *Ahr^fl/fl^* mice; however, H&E-stained liver sections show no apparent difference in hepatic morphology. (**C**) Female cCKO mice challenged with a high-fat diet (HFD) for 15 weeks exhibit decreased weight gain relative to *Ahr^fl/fl^* mice (*n* = 3 per genotype). (**D**) Gross morphology reveals extensive adiposity in HFD-fed female *Ahr^fl/fl^* mice compared to female cCKO mice. Additionally, livers (arrows, inset) in *Ahr^fl/fl^* mice presented with evidence of steatosis. (**E**) H&E-stained gWAT from HFD-fed female cCKO mice demonstrates reduced adipocyte size relative to *Ahr^fl/fl^*, while H&E-stained female *Ahr^fl/fl^* liver sections exclusively, exhibit steatosis. (**F**) H&E-stained gWAT and liver sections from 10-week old male *Ahr^fl/fl^* and cCKO mice exhibit similar morphology. (**G**) Male *Ahr^fl/fl^* and cCKO mice (*n* = 4 per genotype) maintained on HFD for eight weeks do not exhibit any significant change in weight gain. All microscopy images are shown at 200× magnification and representative of three animals. Areas enclosed by a dashed rectangle have been digitally zoomed and shown in the corresponding inset for clarity. Numerical data are presented as mean ± SEM. Statistical analysis was performed using a Student’s *t* test or two-way ANOVA with Bonferonni posttests. Significant differences are notated as * *p* < 0.05, ** *p* < 0.01, or *** *p* < 0.001.

Previous studies demonstrate that a global loss of *Ahr* expression or function reduces body mass and protects against high-fat diet (HFD) challenge [17,18]. However, global AHR loss affects numerous physiological processes including innate immunity, liver function, and the development of the reproductive organs, which confounds efforts to draw conclusions about the organ-specific effects of AHR loss [19,20]. Therefore, we utilized the constitutive, hepatocyte-targeted AHR knockout mouse model to characterize the role of hepatic AHR in metabolic homeostasis for this study. Body mass of female cCKO mice maintained on a standard rodent chow was a significant 11% lower than of *Ahr^fl/fl^* controls at 10 weeks of age (Figure 1A). This difference in body mass was associated with decreased adipocyte size and increased multilocular lipid droplet formation in gWAT, without any visible alteration of liver morphology (Figure 1B). We subsequently challenged female *Ahr^fl/fl^* and cCKO mice with a HFD for 15 weeks, and observed that HFD-fed female cCKO mice gain body mass at a significantly slower rate than *Ahr^fl/fl^* controls (*Ahr^fl/fl^* slope: 0.054 ± 0.003 week^−1^, cCKO slope: 0.022 ± 0.001 week^−1^, *p* < 0.0001) (Figure 1C). Gross morphological assessment further revealed substantially larger white adipose tissue deposits in the *Ahr^fl/fl^* mice, and liver discoloration indicative of steatosis (Figure 1D). Consistent with previous observations, adipocytes within gWAT collected from HFD-fed female cCKO mice are multilocular and visibly smaller than the unilocular fat cells observed in *Ahr^fl/fl^* females (Figure 1E). Histological analysis confirmed steatosis in the livers from HFD-fed female *Ahr^fl/fl^* mice, and not in the livers from female cCKO mice. In contrast to female mice, male *Ahr^fl/fl^* and cCKO mice maintained on standard rodent chow did not exhibit differences in adiposity or any overt changes to gWAT or liver morphology (Figure 1F). Male *Ahr^fl/fl^* and cCKO mice likewise exhibited comparable weight gain during an eight-week HFD challenge (*Ahr^fl/fl^* slope: 0.028 ± 0.003 week^−1^, cCKO slope: 0.035 ± 0.004 week^−1^, *p* = 0.237) (Figure 1G). These data demonstrate that the effects of hepatic AHR loss on adiposity and weight gain are sexually dimorphic. Given the lack of a strong phenotypic difference between *Ahr^fl/fl^* and cCKO male mice, we limited subsequent studies to female mice.

### 2.2. Induced Loss of Hepatocyte AHR Reduces Weight Gain and Adiposity, and Increases Multilocular Lipid Droplet Formation in gWAT.

**Figure 2:**
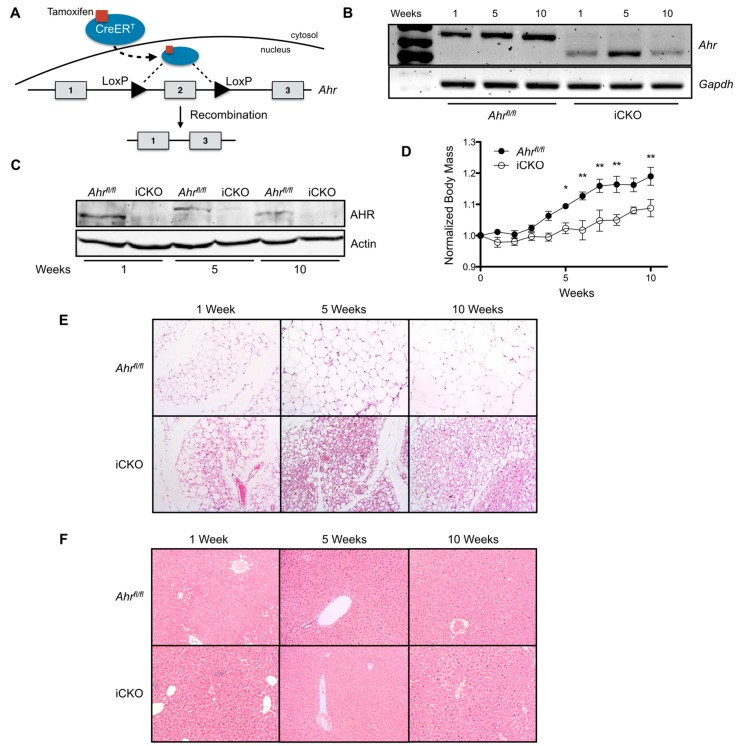
Induced loss of hepatocyte AHR expression in adult female mice reduces weight gain and adiposity. (**A**) Schematic outline of tamoxifen-induced AHR loss. Briefly, tamoxifen-activated *Cre* recombinase (Cre-ER^T2^) excises exon 2 from the genome, resulting in a truncated non-coding *Ahr* transcript. (**B**) Polymerase chain reaction (PCR) monitoring of *Ahr* transcript levels demonstrates exon 2 excision in tamoxifen-treated, inducible AHR conditional knockout mouse livers (*Ahr^fl/fl^Alb-Cre^ERT2^*, abbreviated “iCKO”). (**C**) Tamoxifen treatment results in the sustained loss of hepatic AHR protein expression in iCKO mice. (**D**) Tamoxifen-induced AHR loss reduces weight gain in female iCKO mice maintained on standard rodent chow for 10 weeks (*n* = 6–7 per genotype). (**E**) H&E-stained gWAT collected from female iCKO mice at 1, 5, and 10 weeks after tamoxifen administration show decreased adipocyte size and increased multilocular lipid droplet formation. (**F**) H&E-stained liver sections from female *Ahr^fl/fl^* and iCKO mice at 1, 5, or 10 weeks post-tamoxifen treatment exhibit similar morphology. Protein data are representative of all mice at each time point shown. All microscopy images are shown at 200× magnification and representative of three animals. Numerical data are presented as mean ± SEM. Statistical analyses were performed using a Student’s *t* test or two-way ANOVA with Bonferonni posttests. Significant differences are notated as * *p* < 0.05 or ** *p* < 0.01.

A major limitation to utilizing the cCKO mouse model is that AHR deletion occurs during gestation, which may hinder the ability to differentiate between the primary effects of AHR loss and those that are potentially secondary to the effects of hepatocyte AHR loss on growth and development. Therefore, we generated a novel, tamoxifen-inducible AHR conditional knockout mouse model (iCKO) to further investigate the role of AHR loss in reduced body mass and adiposity in fully developed adult mice. iCKO mice expressed a modified *Cre* recombinase that remained inactive until tamoxifen induces the timed removal of AHR expression in adult mice (Figure 2A). Single-dose treatment with 75 µg/kg tamoxifen for three consecutive days resulted in a sustained loss of functional AHR transcript and protein at 1, 5, and 10 weeks post-treatment (Figure 2B,C). Figure 2D shows that female iCKO mice gained body mass at a significantly reduced rate relative to *Ahr^fl/fl^* controls (*Ahr^fl/fl^* slope: 0.0176 ± 0.0008 week^−1^, iCKO slope: 0.0088 ± 0.0009 week^−1^, *p* < 0.0001), when maintained on a standard rodent chow for 10 weeks after tamoxifen administration. Histological assessment of gWAT from female iCKO mice sacrificed at 1, 5, or 10 weeks post-tamoxifen treatment reveals a distinct reduction of adiposity and increase in multilocular lipid droplet formation compared to *Ahr^fl/fl^* mice (Figure 2E). In contrast, hepatocyte AHR deficiency does not appear to influence liver morphology throughout the 10-week time course (Figure 2F).

### 2.3. Induced AHR Loss Increases Hepatic FGF21 Production and BAT/gWAT Respiratory Capacity, and Is Associated with Greater Energy Expenditure and Water Intake in the Absence of Increased Physical Activity

**Figure 3:**
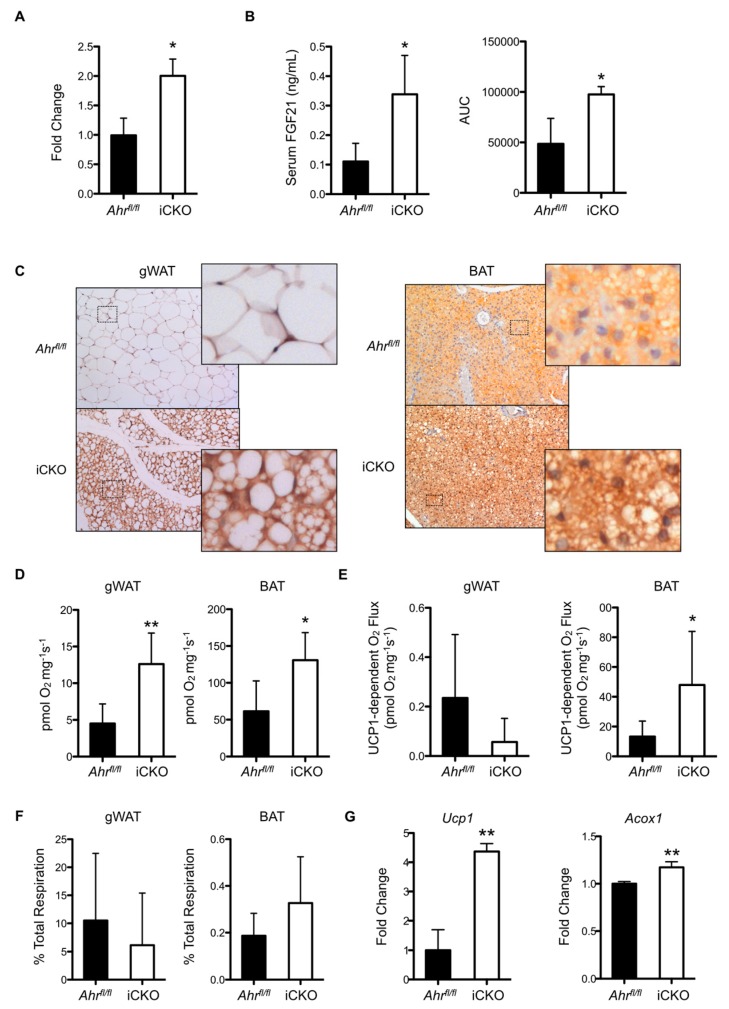
Tamoxifen-induced loss of hepatic AHR expression is associated with increased hepatic FGF21 production and gWAT/ brown adipose (BAT) thermogenesis. (**A**) *Fgf21* mRNA and (**B**) serum FGF21 concentrations as measured by ELISA (left) or Selected Reaction Monitoring mass spectrometry (right) are elevated in iCKO mice (*n* = 3–5 per genotype) at 1 week post-tamoxifen administration. (**C**) Immunohistochemical staining for uncoupling protein 1 (UCP1) is more intense in gWAT and BAT from iCKO mice at 1 week post-AHR loss, and reveals increased multilocular lipid droplet formation. (**D**) Total respiratory capacity in gWAT and BAT is significantly increased in iCKO mice five weeks after tamoxifen treatment (*n* = 6 per genotype). (**E**) Absolute UCP1-dependent respiration is modestly lower in gWAT, but significantly greater in BAT. (**F**) UCP1-dependent respiration as a percentage of total respiration. (**G**) iCKO mice exhibit significantly greater *Ucp1* and *Acox1* gene expression at five weeks post-tamoxifen treatment. All microscopy images are shown at 200× magnification and are representative of three animals. Areas enclosed by a dashed rectangle have been digitally zoomed and shown in the corresponding inset for clarity. Numerical data are presented as mean ± SEM. Statistical analyses were performed using a Student’s *t* test, with significant differences indicated as * *p* < 0.05 or ** *p* < 0.01.

To examine why hepatic AHR deletion consistently results in reduced adiposity and increased multilocular lipid droplet formation in gWAT, we conducted a deep sequencing transcriptomic analysis on liver RNA isolated from *Ahr^fl/fl^* and iCKO livers at one week post-tamoxifen treatment. We chose to focus on the expression of genes involved in the liver-adipose signaling axis because of the targeted deletion of AHR to the liver and the phenotype observed in gWAT. Table 1 highlights several significant changes in hepatokine expression. In agreement with previous data showing that AHR loss increases *Fgf21* transcription [3], we observed an approximate two-fold increase in the expression of *Fgf21* in iCKO mice. Subsequently, we validated this observation using quantitative polymerase chain reaction (PCR) analysis (Figure 3A). Circulating FGF21 protein concentrations were also significantly elevated two- to three-fold in iCKO mice relative to *Ahr^fl/fl^* controls, as measured by both ELISA and a novel Selected Reaction Monitoring mass spectrometry methodology (Figure 3B). Consistent with the observed increase in hepatic FGF21 production and the known functions of FGF21, immunohistochemical staining for UCP1 was enhanced in gWAT tissue from iCKO mice, and revealed an increase in the formation of multilocular lipid droplets relative to floxed controls (Figure 3C). Brown adipose tissue deposits also exhibited enhanced UCP1 staining in iCKO mice relative to *Ahr^fl/fl^*. At five weeks post-tamoxifen administration, the respiratory capacity of permeabilized gWAT tissue from iCKO mice was a significant 2.8-fold greater than in *Ahr^fl/fl^* mice (Figure 3D). Respiratory capacity also significantly increased 2.1-fold in brown adipose tissue (BAT) isolated from iCKO mice relative to *Ahr^fl/fl^*. UCP1-dependent respiration was modestly lower in gWAT, but a significant 3.6-fold greater in BAT (Figure 3E). Figure 3F shows that the ratio of UCP1-associated respiration to total respiration is not statistically different between gWAT isolated from *Ahr^fl/fl^* and iCKO mice, which indicates that AHR loss does not meaningfully alter UCP1 functionality in this tissue. This ratio is slightly elevated in BAT from iCKO, consistent with the significant increase in UCP1-dependent respiration (Figure 3B), albeit falls short of statistical significance (*p* = 0.15). Despite no apparent change in UCP1 function in iCKO gWAT, *Ucp1* transcript remained elevated 4.3-fold at five weeks post-tamoxifen administration (Figure 3G). A significant 17% increase in acyl-CoA Oxidase 1 (*Acox1*) gene expression relative to *Ahr^fl/fl^* further suggests elevated turnover of lipids within gWAT deposits from iCKO mice at this timepoint.

Values are shown as normalized read counts (*n* = 3 per genotype). Abbreviations used: *Angptl3*, angiopoietin-like protein 3; *Ahsg*, alpha-2-HS-glycoprotein; *Fgf21*, fibroblast growth factor 21; *Igfbp1*, insulin-like growth factor binding protein 1; *Igfbp2*, insulin-like growth factor binding protein 2.

**Figure 4:**
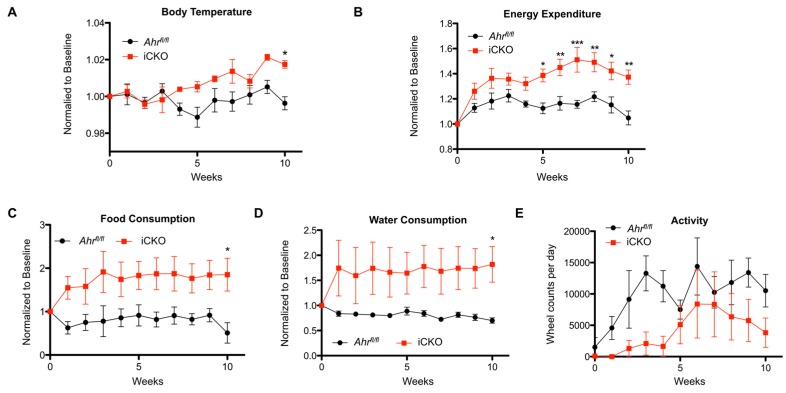
iCKO mice exhibit increased energy expenditure without an increase in physical activity. (**A**) iCKO mice exhibit elevated body temperatures by four weeks following loss of AHR expression, concomitant with a significant increase in (**B**) energy expenditure. (**C**) Food and (**D**) water consumption are dramatically increased in the iCKO mice within the first week following loss of hepatic AHR. (**E**) iCKO mice display a general trend towards reduced physical activity. Numerical data are presented as mean ± SEM of four animals from each genotype. Statistical analyses were performed using two-way ANOVA with Bonferonni posttests. Significant differences are indicated as * *p* < 0.05, ** *p* < 0.01, or *** *p* < 0.001.

Next, we used the non-invasive Comprehensive Laboratory Animal Monitoring System (CLAMS™, Columbus Instruments, Columbus, OH, USA) to simultaneously monitor food and water intake, physical activity, core body temperature, and to perform indirect calorimetry and determine energy expenditure. *Ahr^fl/fl^* and iCKO mice were continuously monitored over a 24 h time period, once per week, for 10 weeks. The data presented in Figure 4A–E encompass the 12-h dark cycle when the mice were most active and differences were observed between the genotypes. Consistent with the significant increase of respiratory capacity in gWAT and BAT upon induced AHR loss (Figure 3D), iCKO mice maintained elevated body temperature (*Ahr^fl/fl^* slope: 0.00004 ± 0.0004 week^−1^, iCKO slope: 0.00214 ± 0.0004 week^−1^, *p* = 0.0006) and energy expenditure (*Ahr^fl/fl^* slope: 0.0003 ± 0.0005 week^−1^, iCKO slope: 0.0303 ± 0.0071 week^−1^, *p* = 0.002) (Figure 4A,B). iCKO mice also consumed significantly more food and water; however, they appeared to be less active overall (Figure 4C–E), suggesting that the increased energy expenditure was attributable to increased respiration in gWAT (Figure 3D) and increased thermogenesis in BAT (Figure 3E).

### 2.4. Combinatory Deletion of AHR and FGF21 from Hepatocytes Reverses the Effects of Hepatic AHR Deficiency

**Figure 5:**
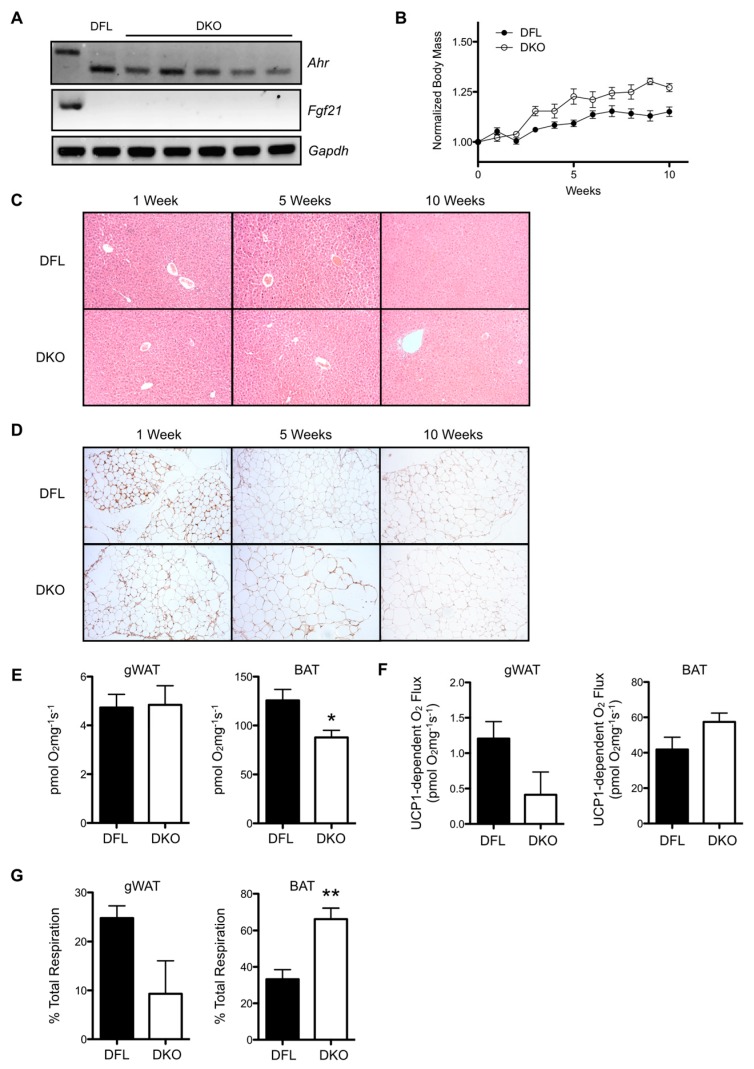
Concomitant loss of AHR and FGF21 reverses the iCKO phenotype. (**A**) *Ahr* and *Fgf21* transcripts are ablated in the livers of inducible, double-conditional AHR-FGF21 knockout mice (*Ahr^fl/fl^Fgf21^fl/fl^Alb-Cre^ERT2^*, abbreviated “DKO”) 10 weeks after tamoxifen administration (*n* = 6). (**B**) DKO mice exhibit greater weight gain over a 10-week period following tamoxifen administration (*n* = 5–6 per genotype). (**C**) H&E staining of liver sections from *Ahr^fl/fl^Fgf21^fl/fl^* (DFL) and DKO mice show comparable liver morphology at 1, 5, and 10 weeks post-tamoxifen treatment. (**D**) UCP1 immunohistochemical staining of gWAT from DFL and DKO mice at 1, 5, and 10 weeks post-tamoxifen treatment reveals no apparent difference in stain intensity or in adipocyte morphology. (**E**) Total respiratory capacity is similar in gWAT from DFL and DKO mice at five weeks, but significantly decreased in BAT from DKO mice. (**F**) Absolute UCP1-dependent respiration in gWAT and BAT. (**G**) UCP1-specific respiration as a percentage of total respiratory capacity. Microscopy images are shown at 200× magnification and are representative of three animals. Numerical data are presented as mean ± SEM. Statistical analysis was performed using a Student’s t test, with statistically significant differences indicated as * *p* < 0.05 or ** *p* < 0.01.

To verify the role of FGF21 in mediating the effects of hepatic AHR deficiency on white adipose tissue morphology and gWAT/BAT respiratory capacity, we generated an inducible, double-conditional AHR-FGF21 knockout (DKO) mouse in which tamoxifen treatment results in simultaneous excision of exon 2 from *Ahr*, and exons 1–3 from *Fgf21*. Figure 5A demonstrates the successful and persistent deletion of *Ahr* and *Fgf21* transcripts in individual mice at 10 weeks post-tamoxifen administration. DKO mice maintained on standard rodent chow exhibit significantly faster weight gain than *Ahr^fl/fl^Fgf21^fl/fl^* (DFL) control mice (DFL slope: 0.016 ± 0.002 week^−1^, DKO slope: 0.030 ± 0.003 week^−1^, *p* < 0.0001) during a 10-week time course (Figure 5B). Consistent with previous data, DFL and DKO mice displayed comparable liver morphology at 1, 5, and 10 weeks post-tamoxifen treatment (Figure 5C). Immunohistochemical staining for UCP1 in gWAT at these time points demonstrates minimal staining in both DFL and DKO mice (Figure 5D). We also observed no visible differences in adipose tissue morphology between the two strains throughout the 10-week period. After five weeks post-AHR loss, total respiratory capacity in gWAT from DFL and DKO mice was near equivalent, while BAT respiratory capacity was reduced by a significant 30% in DKO mice (Figure 5E). Figure 5F shows that absolute UCP1-dependent respiration was modestly decreased in gWAT from DKO mice, and slightly increased in BAT relative to DFL; however, the differences fall short of statistical significance. When calculated as a percentage of total respiratory capacity, UCP1-dependent respiration appears to contribute modestly less to total respiration in DKO mice (*p* = 0.07) (Figure 5G). In contrast, the contribution of UCP1-dependent respiration to total BAT respiration was significantly increased two-fold in DKO mice relative to DFL.

### 2.5. AHR Binding to a Novel XRE Site within the Fgf21 Promoter Is Associated with the Suppression of Fgf21 Transcription

**Figure 6:**
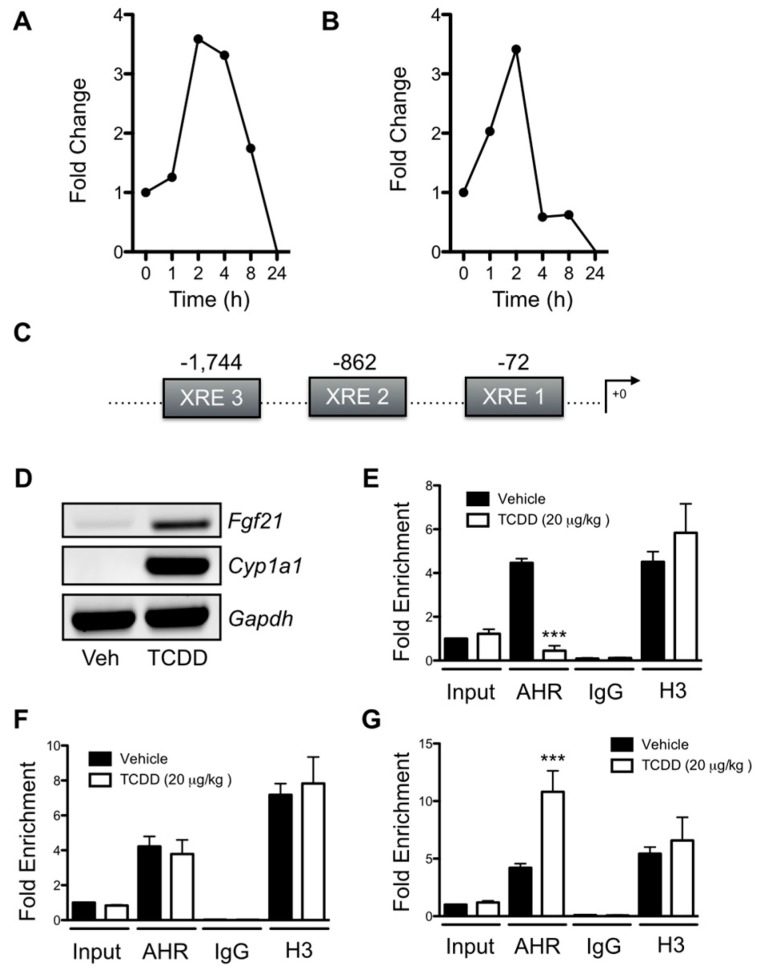
Transient AHR agonist induction of *Fgf21* transcription correlates with distinct binding events at the *Fgf21* promoter. (**A**) 6 nM 2,3,7,8-tetrachlorodibenzo-*p*-dioxin (TCDD) and (**B**) 30 μM cinnabarinic acid transiently induce *Fgf21* transcription in *Ahr^fl/fl^* primary hepatocytes at 2 h, with gene expression subsiding by 24 h. (**C**) Schematic indicating the locations of XRE1, XRE2, and XRE3, within the *Fgf21* promoter. (**D**) Exposure of female *Ahr^fl/fl^* mice (*n* = 3) to 20 μg/kg TCDD induces hepatic *Cyp1a1* and *Fgf21* transcription at 2 h, and is associated with (**E**) reduced AHR binding at XRE3. (**F**) AHR exhibits constitutive, agonist-independent binding at XRE2. (**G**) TCDD exposure in *Ahr^fl/fl^* females increases AHR binding to XRE1 at 2 h. Gene expression is calculated as fold-change from time point zero. Chromatin immunoprecipitation (ChIP) data are calculated as fold enrichment from vehicle input and presented as mean ± SEM. All experiments were performed in triplicate. Statistical analysis was performed using a Student’s *t* test, with significant differences relative to vehicle denoted as *** *p* < 0.001.

Previous studies demonstrate that the AHR-ARNT heterodimer can bind to the *Fgf21* promoter and that AHR agonists can both activate and suppress *Fgf21* transcription [2,3]. However, the relationship between AHR binding events and changes in *Fgf21* gene expression remains unclear. We observed a transient induction of *Fgf21* transcription in primary *Ahr^fl/fl^* hepatocytes exposed to 6 nM TCDD (Figure 6A) or 30 μM cinnabarinic acid (Figure 6B), an endogenous agonist, over a 24 h period, with maximal induction at 2 h and complete suppression by 24 h. We attempted to reconcile AHR binding at the *Fgf21* promoter with these transcriptional changes utilizing chromatin immunoprecipitation (ChIP) assays. In addition to the previously identified XRE within the *Fgf21* promoter (XRE1), we located two additional XRE motifs that potentially play a role in AHR-modulated *Fgf21* transcription (Figure 6C). These binding sites are located 1,744 bp (XRE3) and 862 bp (XRE2) upstream of the transcription start site. Increased *Fgf21* and *Cyp1a1* mRNA was readily detectable in the livers of *Ahr^fl/fl^* mice treated with 20 μg/kg TCDD for 2 h (Figure 6D). ChIP assays performed with whole livers from *Ahr^fl/fl^* mice treated with 20 μg/kg TCDD or vehicle for 2 h demonstrated a decrease in AHR binding at XRE3 relative to vehicle (Figure 6E). In contrast, 2 h TCDD exposure did not alter AHR binding to XRE2; however, it did increase binding to XRE1 (Figure 6F–G).

## 3. Discussion

The hepatocyte-specific conditional AHR knockout mouse model (cCKO) that Christopher Bradfield et al. engineered has become a popular tool for investigating hepatic AHR function [16]. This model disrupts the adaptive capacity of the liver to respond to exogenous halogenated aryl hydrocarbons (e.g., TCDD), without affecting the development of hepatic vasculature. Our data revealed that constitutive, hepatocyte-targeted loss of the AHR results in reduced body mass exclusively in female mice maintained on standard rodent chow. Decreased body mass was associated with reduced adiposity and increased multilocular lipid droplet formation within gWAT, both evidence of the browning of white fat [7]. Remarkably, these sex-specific properties were also observed in cCKO mice maintained on an HFD, as well as in the iCKO mice. Sexual dimorphism in glucose and lipid metabolism, growth, and gene expression profiles are not uncommon in mice and humans [21,22,23]. AHR interference with estrogen receptor signaling has previously been reported through multiple mechanisms of action [24], and therefore, we speculate that cross-talk between these transcription factors may be responsible for the observed sexual dimorphism in the cCKO and iCKO. Nevertheless, we cannot discount other inherent metabolic differences between male and female mice as accounting for the observed sexual dimorphism [25]. Previous data demonstrated that global AHR inhibition or knockdown protects against HFD feeding in male mice [17,18,26]. We believe that the inability to reproduce a protective phenotype in male cCKO or iCKO mice in this study derives from distinct differences in the model systems utilized.

The observation that both cCKO and iCKO mice exhibit reduced weight gain and adiposity phenotype strongly suggests that this is directly attributable to hepatic AHR loss affecting sustained homeostatic processes, rather than an AHR role in liver development. The consistency of findings in both the cCKO and iCKO mice also indicate that the effects observed upon AHR loss are physiologically significant, rather than an artifact. Data generated from the CLAMS experiments establish that the decrease in adiposity in iCKO mice is associated with an increase in energy expenditure, body temperature, and food and water consumption, but not physical activity. These data are consistent with the corresponding increase of gross BAT and gWAT respiratory capacity observed in iCKO mice. Together, the data highlight the importance of AHR activity in systemic physiology, and are consistent with the recognition that the liver constantly communicates with other organs through largely undetermined processes [27].

We utilized deep sequencing to interrogate the liver transcriptome and identify candidate gene targets capable of functioning as hepatokines to impact adipocyte metabolism through the liver-adipose signaling axis. iCKO mice, which exhibit two-fold greater *Fgf21* gene expression relative to *Ahr^fl/fl^*, demonstrated a strikingly similar phenotype to that observed upon pharmacological FGF21 administration: weight loss, increased BAT/gWAT respiration, increased water intake, and increased energy expenditure without an increase in physical activity [10,28]. The data also show that transcription of angiopoietin-like protein 3 (*Angptl3*) was decreased 4.2-fold in iCKO mice. Because *Angptl3* expression positively correlates with the inhibition of lipoprotein lipase activity and reduced fatty acid uptake in adipose, reduced *Angptl3* expression in iCKO mice would predict greater fat accumulation [29,30]. This suggests that *Angptl3* is not a contributor to the reduced fat phenotype. iCKO mice also exhibit a significant 1.6-fold reduction in alpha-2-HS-glycoprotein (*Ahsg*) gene expression. Loss of AHSG in mice has been linked to improved insulin sensitivity and resistance to weight gain [31]. Insulin-like growth factor binding protein 1 (*Igfbp1*) is a target gene of autocrine hepatic FGF21 action and increased circulating IGFBP1 levels are associated with enhanced insulin sensitivity [32,33]. We observed a 3.7-fold increase in the expression of *Igfbp1* in iCKO mice, consistent with the induction of hepatic FGF21 production. Insulin-like growth factor binding protein 2 (IGFBP2) is inversely correlated with fat accumulation in humans, and is expressed two-fold greater in iCKO mice [34]. Although transcriptional shifts in these hepatokines following loss of the AHR are largely consistent with the observed reduced weight gain phenotype, the complete reversal of the phenotype in DKO mice suggests that exclusive of FGF21, these hepatokines are not significant contributors to the effects of AHR loss in cCKO and iCKO female mice.

Employing a unique approach to directly assess UCP1 function and overall mitochondrial respiratory capacity [35,36], we demonstrated that iCKO mice have increased BAT and gWAT respiratory capacity. Despite significantly increased UCP1 transcription and immunohistochemical staining in the gWAT of iCKO mice relative to *Ahr^fl/fl^*, neither absolute UCP1-dependent respiration, nor the ratio of UCP1-dependent respiration to total respiration, was increased. These data suggest that while gWAT from iCKO mice may physically contain more UCP1 protein, this increase in protein does not explain the increase in respiratory capacity. Given the observed increase of *Acox1* mRNA, increased gWAT respiration might instead reflect an increased flux of lipids into the beta-oxidation cycle and subsequent increase in oxidative phosphorylation. In contrast to iCKO mice, DKO mice did not exhibit increased gWAT respiratory capacity relative to control DFL mice. These data support the previously established role of FGF21 in promoting white fat respiration and lipolysis. [10,37]. Absolute UCP1-dependent respiration and its contribution to total respiratory capacity were modestly decreased in DKO mice, implying that FGF21 is necessary for sustained homeostatic UCP1 expression in gWAT. This is consistent with previous studies demonstrating a role for FGF21 in regulating UCP1 expression [5,6]. BAT respiratory capacity is significantly decreased in DKO mice, which suggests that liver-excreted FGF21 protein might also play a role in sustaining basal BAT respiration. Although BAT respiratory capacity is diminished in DKO mice, we observed an increase in UCP1 function. However, this increase does not appear to correct for an overall reduction of BAT respiratory capacity, as DKO mice still exhibit greater weight gain relative to DFL. Increased UCP1 function in BAT from DKO predicts the existence of one or more compensatory mechanisms for activating UCP1, despite the loss of hepatic FGF21 signaling. Indeed, several mechanisms of stimulating UCP1 function in BAT have been previously described, including the release of norepinephrine, insulin pathway signaling, and circulating thyroid hormones [38,39]. Additional studies are required to determine which of these may be a contributing factor to the observed increase of UCP1 function in BAT from DKO mice. The observation that FGF21 loss does not result in a loss of thermogenic function in BAT also adds to the accumulating evidence that FGF21 may not be required for UCP1 function within BAT [40]. One recent review suggests that FGF21 can even exert UCP1-independent action [41]. Given the combined DKO phenotype of enhanced weight gain, a reduction in gWAT UCP1 function, and a marked reduction of BAT respiratory capacity, we speculate that basal, constitutive *Fgf21* expression under AHR control in wild-type mice may temper fat accumulation and help sustain homeostatic UCP1 function in gWAT and BAT. Alternatively, this phenotype may highlight the increased involvement of other transcription factors that influence metabolic homeostasis via regulating hepatic *Fgf21* expression in the absence of AHR, such as the peroxisome proliferator activated receptor α, the retinoic acid receptor β, and the carbohydrate response element-binding protein [42,43,44]. FGF21 has also been shown to regulate metabolism via acting upon the nervous system [45]; however, our data cannot distinguish between the direct FGF21 effects on adipocytes and FGF21 signaling through the central nervous system to control chronic metabolism.

Comparable to the data presented in this study, Lu et al. (2015) previously showed that the expression of constitutively-active human AHR (CA-AHR) protein within mouse livers can partially protect against HFD challenge through the induction of *Fgf21* gene expression. The authors specifically utilized shRNA-mediated knockdown of FGF21 to demonstrate that FGF21 was required for these AHR-driven effects. CA-AHR expression produced a notably similar phenotype to that observed upon AHR loss in the current study, i.e., reduced adipocyte size within brown and white fat deposits and increased thermogenic activity. In a separate study employing *Ahr^+/−^* heterozygous mice, the partial loss of global AHR expression was also associated with elevated *Ucp1* expression in BAT and skeletal muscle, and increased energy expenditure in the absence of increased locomotor activity, akin to the data presented here [18]. The ability of both reduced AHR expression and constitutive AHR activity to activate *Fgf21* transcription may appear paradoxical; however, similar discordant findings involving AHR activity have been previously observed. Specifically, both loss of the AHR and its prolonged activation can slow cell proliferation [46], suggesting that receptor-mediated biological processes are very sensitive to AHR status. We postulate that the AHR plays a critical role in maintaining *Fgf21* transcriptional homeostasis; whereas the loss of hepatic AHR expression negates its physiologic inhibitory role and permits the constitutive activation of *Fgf21* gene expression via other transcription factors, the presence of canonical XREs within the *Fgf21* promoter still allows for AHR ligand-driven gene transcription.

AHR agonists can both activate and suppress *Fgf21* transcription; however, the precise mechanism behind the bidirectional AHR control over *Fgf21* transcription remains unclear. Our data indicate that AHR agonist-mediated induction of *Fgf21* gene expression is transient, with robust *Fgf21* expression correlating with increased agonist-dependent binding of AHR at the proximal XRE (XRE1), and concomitant loss of AHR binding at the distal XRE (XRE3). This suggests that AHR binding at XRE1 promotes transcriptional activity, while binding at XRE3 may suppress *Fgf21* transcription in an agonist-dependent manner under normal physiological conditions. The role of XRE2 in *Fgf21* expression remains unknown given the observed constitutive and unaltered AHR recruitment to that site. Ongoing efforts to recapitulate these *Fgf21* promoter-binding properties using ectopic reporter expression systems have proven unsuccessful, suggesting that bona fide chromatin architecture plays a critical role in the transcriptional response to AHR ligand. Therefore, future studies will seek to reconcile these AHR binding events with functional changes in *Fgf21* transcription by utilizing CRISPR/Cas9 gene editing to introduce targeted mutations into the XREs that disrupt AHR binding.

In summary, our data demonstrate that induced AHR loss in adult female mice activates BAT thermogenesis and gWAT respiration through increased hepatic release of FGF21. FGF21 has been actively assessed for its therapeutic potential to treat metabolic disease due to its success in counteracting obesity in several different animal models. However, wildtype FGF21 protein was previously found unsuitable for therapeutic applications, due to various biopharmaceutical issues such as conformational instability [14]. An engineered FGF21 variant, LY2405319, has produced promising results in obese human subjects, but remains a controversial option due to high dosing requirements [47]. The AHR might therefore be an attractive ‘druggable’ target for FGF21-based therapies due to the multiple methods by which the receptor can increase hepatic FGF21 production. Current work in the laboratory is being conducted on screening endogenous AHR ligands for their ability to regulate *Fgf21* transcription.

## 4. Materials and Methods

### 4.1. Animals and Treatments

All animal experiments utilized eight- to 10-week old mice. *Ahr^fl/fl^* (strain designation B6.129S-*Ahr^tm3.1Bra^*/J), *Alb-Cre* (strain designation B6.Cg-Tg(Alb-cre)21Mgn_J), and *Fgf21^fl/fl^* (strain designation B6.129S6(SJL)-*Fgf21^tm1.2Djm^*/J) were purchased from The Jackson Laboratory (Bar Harbor, ME). Mice expressing a modified, inducible *Cre* recombinase (strain designation B6.Alb-Cre-ER^T2^) were obtained from Ben Strangers (University of Pennsylvania, Philadelphia, PA, USA) with permission from Pierre Chambon (Pasteur Institute, Paris, France) [48]. To generate an inducible AHR knockout mouse model, B6.Alb-Cre^ERT2^ mice were crossed with C57BL6/J-back-crossed *Ahr^fl/fl^* mice. C57BL6/J-back-crossed *Ahr^fl/fl^* and *Fgf21^fl/fl^* were then bred together to generate double-floxed mice (*Ahr^fl/fl^Fgf21^fl/fl^*, “DFL”). To generate double-inducible AHR-FGF21 knockout mice, DFL mice were crossed to B6.Alb-Cre^ERT2^ mice. Animals were maintained on either a standard rodent chow (Harland Tekland #7912, Madison, WI, USA) or a 60% kcal high-fat diet (Research Diets #D12079B, New Brunswick, NJ, USA), and housed on corn cob bedding in a pathogen-free, climate- and temperature-controlled facility. Food and water were provided ad libitum. No significant differences in food intake between strains were observed in any experiment. To induce gene deletions, Alb-Cre^ERT2^-expressing mice were administered a single dose of tamoxifen (75 µg/kg) by gavage for three consecutive days. To control for any potential tamoxifen-mediated effects, floxed animals also received these treatments. All mice were fasted for 5 h prior to sacrifice. Upon sacrifice, sections of gWAT and liver were snap-frozen in liquid nitrogen and stored at −80 °C. All mouse experiments were conducted humanely and in accordance with the Animal Care and Use Committee Guidelines at the University of Texas Medical Branch at Galveston (Protocol #0109034E, Approved 9 January 2016) and Guide for the Care and Use of Laboratory Animals as adopted and promulgated by the USA National Institutes of Health.

### 4.2. RNA Extraction, Semi-Quantitative PCR, and Transcriptomic Analyses

RNA was isolated utilizing Trizol Reagent as per manufacturer’s instructions (Life Technologies, Carlsbad, CA). cDNA was prepared as previously described [49]. PCR was performed utilizing Taq polymerase (Fisher Scientific, Pittsburgh, PA, USA) and the following sets of primers: *Acox1,* Forward: 5′ CAGGAAGAGCAAGGAAGTGG 3′, Reverse: 5′ CCTTTCTGGCTGATCCCATA 3′; *Ahr,* Forward: 5′ CGCAAGCCGGTGCAGAAAAC 3′, Reverse: 5′ ATGGAGGGTGGCTGAAGTGGAGTA 3′; *Fgf21*, Forward: 5′ GGGGATTCAACACAGGAGAA 3′, Reverse, 5′ AGGGCCTCAGGATCAAAGTGA 3′; *Gapdh*, Forward: 5′ ACGGCAAATTCAACGGCACAGTCA 3′, Reverse: 5′ CATTGGGGGTAGGAACACGGAAGG 3′; *Ucp1* Forward: 5′ AGGATGGTGAACCCGACAAC 3′, Reverse: 5′ CCGAGAGAGGCAGGTGTTTC 3′. For transcriptomic analyses, RNA samples were reverse transcribed into cDNA, then fragmented and amplified using paired-end primers on an Illumina HiSeq 1000 sequencing system at the UTMB Genomics Core Facility to evaluate differences in liver transcripts. A *p*-value cutoff of 0.05 was utilized to identify differentially expressed gene transcripts from the dataset, as calculated using the tophat-cufflink-cuffdiff suite of analysis programs (UTMB Genomics Core Facility, Galveston, TX, USA).

### 4.3. Western Blot Analysis of AHR.

Frozen liver samples (−80 °C) were homogenized in cell lysis buffer (Cell Signaling Technology, Danvers, MA, USA) utilizing a Brinkman polytron homogenizer (Kinematica AG, Lucerne, Switzerland). Protein concentration was measured using a modified Lowry assay (Bio-Rad, Hercules, CA, USA). Samples were then diluted in 2× SDS loading buffer + 10% 2-mercaptoethanol (BME) and boiled for five minutes. 30 µg of each sample was loaded onto a 10% SDS-polyacrylamide gel and separated at 120 V for 120 min, then transferred onto a polyvinylidene fluoride (PVDF) membrane (Bio-Rad, Carlsbad, CA, USA). Membranes were blocked utilizing Tris-buffered saline (TBS) containing 0.1% (*v*/*v*) Tween 20 and 5% (*w*/*v*) nonfat dry milk, then incubated overnight with polyclonal AHR antibody (BML-SA210, Enzo Life Sciences, Inc., Farmingdale, NY, USA) or monoclonal Actin antibody at 4°C. After washing with TBS, membranes were incubated in the presence of fluorescent secondary antibodies (Abcam, Cambridge, MA, USA) for 1 h at room temperature and visualized on a Typhoon Trio Variable Mode Imager (GE Healthcare, Chicago, IL, USA).

### 4.4. Serum FGF21 Analyses

Blood was collected via cardiac puncture, incubated at room temperature for 30 min, and then centrifuged at 5000× *g* for 10 min to obtain serum. FGF21 was assayed using a Mouse/Rat FGF21 ELISA kit (Biovendor, Asheville, NC, USA) and via the selected reaction monitoring methodology described in Section 4.5 and Section 4.6.

### 4.5. Tryptic Digest of Serum Aliquots

Serum aliquots containing 250 µg of protein were brought to a total volume of 100 µl with 50 mM ammonium bicarbonate buffer. 10 µL of 8 M GdmCl was added and samples were mixed well, then incubated for 30 min at room temperature. 10 µL of 0.1 M DTT was then added and tubes were incubated for an additional 30 min at room temperature. Next, 11 µL of 0.3 M iodoacetamide was added and samples were incubated for 2 h at 37 °C. Samples were briefly centrifuged to collect water, and trypsin added at a ratio of 1:50 trypsin:protein. Then, 889 µL of 50 mM ammonium bicarbonate was added, and samples were subsequently incubated for 24 h at 37 °C on a shaker. Tryptic digestion was deactivated by incubating samples for 10 min at 95 °C on a sample heating block. Finally, 12.1 µL of 0.1% trifluoroacetic acid (TFA) was added to samples prior to desalting with a Sep Pak C-18 column (Waters, Taunton, Massachusetts, MA, USA), as per manufacturer’s protocol. Samples were eluted from the column using 80% ACN, dried overnight in a speed vacuum, then resuspended in 0.1% formic acid and stored at 4 °C prior to analysis.

### 4.6. Selective Reaction Monitoring (SRM)

To develop the SRM method, unique peptides specific to FGF21 were identified using a BLAST search of the mouse FGF21 sequence on the ExPASy portal (Swiss Institute of Bioinformatics, Switzerland). We chose three unique peptides (DSPNQDATSWGPVR, ALKPGVIQILGVK, and SPESLLELK), then cross-validated their uniqueness and identified precursors/product ions and their respective charge states and transitions using Skyline software (MacCoss Software, Seattle, WA, USA). LC-SRM-MS was performed using a triple quadrupole mass spectrometer as previously described [50,51]. SRMs for the three unique peptides were performed using three different transitions for each peptide, with each transition having one quantifier and two qualifiers. The raw data were processed with Skyline to visually inspect the traces of the SRM data and to calculate the peak heights of transitions. One quality transition per peptide was chosen to be used for further analysis. Peak heights of the three proteotypic FGF21 peptides were normalized to an internal standard, and the relative abundance of each peptide across samples was determined using the median peak height of the internal standards. For the generation of area under the curve calculations, published R scripts were used. Other statistical analyses were performed using GenStat (Version 0.15, VSN International, Hemel Hempstead, UK).

### 4.7. Histological Analyses

Liver, BAT, and gWAT were fixed in PBS + 10% formalin for 24 h, then stored in 70% ethanol. Sections were professionally prepared and stained with hematoxylin and eosin (H&E) or subjected to immunohistochemical staining for UCP1 (Vel Lab Research, Missouri City, TX, USA). Single-blind, randomized images were obtained using a Zeiss HAL100 microscope equipped with an Axiocam 512 color camera, operated via ZEN 2.3 software (Carl Zeiss Microscopy GmbH, Munich, Germany).

### 4.8. High-Resolution Respirometry

Respirometry measurements were made as previously described [35]. Briefly, gWAT, and BAT sections were collected immediately upon sacrifice and then submerged in cold modified ethylene glycol-bis(β-aminoethyl ether)-*N*,*N*,*N*′,*N*′-tetraacetic acid (EGTA) buffer. Samples were then blotted on filter paper, dried, and placed into an Oxygraph-2k respirometer to measure respiration.

### 4.9. Comprehensive Lab Animal Monitoring System

The Comprehensive Lab Animal Monitoring System (Columbus Instruments, Columbus, OH, USA), abbreviated “CLAMS”, was utilized to simultaneously measure several metabolic parameters in *Ahr^fl/fl^* and iCKO mice (*n* = 4 per genotype). Physical activity was quantified through wheel counts on a treadmill. Body temperature was monitored with a surgically-implanted telemetry probe. Indirect calorimetry was determined based on respiratory gas exchange (oxygen consumption and CO_2_ production) using the Oxymax™ system. Energy expenditure was calculated by aligning indirect calorimetry data to the feeding and activity habits of the matching animal. Baseline measurements were obtained one week after surgical insertion of the telemetry probe. Animals were then transferred to standard housing conditions and administered a single dose of 75 µg/kg tamoxifen on three consecutive days. Weekly thereafter, animals were transferred to individual CLAMS cages for a 24 h period and then returned to standard housing conditions. During the 24 h period in CLAMS caging, measurements were obtained every 18 min. All data except for wheel counts were normalized to the baseline measurement.

### 4.10. Statistical Analyses

Numerical data are presented as mean ± SEM. Rate of change of body mass, body temperature, and energy expenditure was calculated utilizing linear regression in Graphpad Prism Software V5.0. Where mentioned in the text, ANCOVA was utilized to determine if two slopes were different from one another. A two-tailed Student’s *t* test or two-way ANOVA with Bonferonni posttests was utilized to identify significant differences between groups. All data are representative of two or more independent experiments.

## Figures and Tables

**Table 1:** Changes in hepatokine expression between *Ahr^fl/fl^* and iCKO mice, 1 week after tamoxifen treatment.

Gene	*Ahr^fl/fl^*	iCKO	Fold Change	*p* Value	*q* Value
*Angptl3*	417.2	98.30	−4.243	0.00005	0.0004340
*Ahsg*	6757	4283	−1.578	0.00505	0.0241456
*Fgf21*	0.788	1.520	1.929	0.00265	0.0140155
*Igfbp1*	59.58	219.7	3.687	0.00005	0.0004340
*Igfbp2*	258.2	514.8	1.994	0.00005	0.0004340

## Abbreviations

ACOX1	Acyl-CoA Oxidase 1
AHR	Aryl hydrocarbon receptor
AHSG	Alpha-2-HS-glycoprotein
ANTGL3	Angiopoietin-like protein 3
ARNT	Aryl hydrocarbon receptor nuclear translocator
BAT	Brown adipose tissue
CYP1A1	Cytochrome P450 1A1
GWAT	Perigonadal white adipose tissue
IGFBP1/2	Insulin-like growth factor binding protein 1/2
FGF21	Fibroblast growth factor 21
SRM	Selective reaction monitoring
TCDD	2,3,7,8-tetrachlorodibenzo-*p*-dioxin
UCP1	Uncoupling protein 1
XRE	Xenobiotic response element
